# Management of Breakthrough Bleeding During Emicizumab Prophylaxis in Acquired Haemophilia A: Data From the GTH‐AHA‐EMI Study

**DOI:** 10.1111/hae.70339

**Published:** 2026-06-15

**Authors:** Halet Türkantoz, Christiane Dobbelstein, Richard Greil, Christina Hart, Robert Klamroth, Paul Knöbl, Johannes Oldenburg, Christian Pfrepper, Ulrich Sachs, Inga M. Schimansky, Karolin Trautmann‐Grill, Andreas Tiede

**Affiliations:** ^1^ Hematology, Haemostasis, Oncology, and Cell Therapy Hannover Medical School Hannover Germany; ^2^ IIIrd Medical Department, Salzburg Cancer Research Institute CCCIT; Cancer Cluster Salzburg Paracelsus Medical University Salzburg Salzburg Austria; ^3^ Department of Hematology and Oncology University Hospital Regensburg Regensburg Germany; ^4^ Internal Medicine, Vascular Medicine and Coagulation Disorders Vivantes Clinic Friedrichshain Berlin Germany; ^5^ Department of Medicine 1, Div. Hematology and Hemostasis Medical University of Vienna Vienna Austria; ^6^ Institute of Experimental Hematology and Transfusion Medicine University Hospital Bonn, Medical Faculty, University of Bonn Bonn Germany; ^7^ Division of Haemostaseology, Medical Department I University Hospital Leipzig Leipzig Germany; ^8^ Department of Thrombosis and Haemostasis Giessen University Hospital Giessen Germany; ^9^ Medical Clinic I University Hospital Carl Gustav Carus, TU Dresden Dresden Germany; ^10^ Clinical Chemistry and Laboratory Medicine Hannover Medical School Hannover Germany

**Keywords:** emicizumab, factor 8 deficiency acquired, haemorrhage, recombinant factor VIIa, safety, tranexamic acid

## Abstract

**Introduction:**

The GTH‐AHA‐EMI study showed that emicizumab reduces bleeding in patients with acquired haemophilia A (AHA). However, 22 clinically relevant new bleeds (CRNB) occurred in 14 of the 47 study patients, most of which required haemostatic treatment.

**Aim:**

To describe characteristics, treatment, and outcome of CRNB that occurred during the 12 weeks of haemostatic prophylaxis with emicizumab.

**Methods:**

Data were extracted from the GTH‐AHA‐EMI study data base. Further information was collected retrospectively using a standardized questionnaire answered by study investigators.

**Results:**

Of the 22 CRNB, 9 and 13 were classified as major and non‐major, respectively. 15 bleeds occurred spontaneously, and 20 bleed required treatment. Recombinant factor VIIa was used for treatment in 16 of 20 treated bleeds, recombinant porcine factor VIII was used in three events. Eight patients were admitted to the hospital because of CRNB; extension of hospitalization was reported in 13 patients; 9 were admitted to an intensive care unit; invasive interventions were required in nine patients. Most bleeds were treated successfully; permanent harm from bleeding was reported in two patients. No thromboembolic events or other adverse events were reported.

**Conclusion:**

Although emicizumab reduces bleeding in AHA, breakthrough bleeding can still occur, was often severe and required haemostatic treatment. Haemostatic treatment was safe and usually effective.

**Trial Registration:**

NCT04188639

## 1 Introduction

Acquired haemophilia A (AHA) is a rare bleeding disorder that is characterized by autoantibodies against coagulation factor VIII (FVIII), called inhibitors, that arise in individuals without a history of bleeding [1, 2]. While 50%–70% of cases are idiopathic, an association with malignancy, other autoimmune diseases, and the postpartum period has been described [3, 4, 5].

Since patients with newly diagnosed AHA often present with severe bleeding, that occurs spontaneously or trauma‐induced, immediate bleed control is usually the priority [6]. Frequently used agents are recombinant activated factor VII (rFVIIa, eptacog alfa activated), recombinant porcine factor VIII (rpFVIII, susoctocog alfa), or activated prothrombin complex concentrate (APCC) [6, 7]. These agents are administered intravenously in the setting of acute bleeding and require continuous medical supervision by experienced physicians in the hospital.

Emicizumab, a bispecific monoclonal antibody mimicking the activity of factor VIII, was recently introduced into the management of AHA. Objectives to use emicizumab in AHA include 1) Prevention of bleeds, 2) Reduction of the need for rFVIIa, APCC, and rpFVIII, and 3) Decreases the need for immediate and aggressive immunosuppression [8].

The use of emicizumab in AHA is supported by several case series [9] and two clinical trials: the GTH‐AHA‐EMI study performed in Austria and Germany (*n* = 47, [10]) and the AGEHA study performed in Japan (*n* = 14, [11]).

GTH‐AHA‐EMI was a multicentre, open‐label phase 2 study that assessed the ability of emicizumab to prevent bleeding while immunosuppressive therapy was postponed and most patients remained at active AHA with very low FVIII activity [10]. The bleeding rate during the first 12 weeks of prophylaxis with emicizumab was 68% lower than the historic rate documented in patients treated with immunosuppressive therapy [12]. After two years of continued observation, overall survival was significantly improved (hazard ratio for mortality 0.39; 95% confidence interval, 0.19–0.80), corresponding to an absolute survival difference of 19 percentage points. Only one patient died from infection [13]. Breakthrough bleeding events occurring in the study were not related to baseline clinical characteristics [14] but to the peak of thrombin generation measured after emicizumab loading was complete [15].

If clinically relevant new bleeds (CRNB) occur despite prophylaxis with emicizumab, they may require additional haemostatic treatment. The safety and efficacy of this treatment deserve to be studied, as bleeding in AHA is often different from congenital haemophilia with respect to bleed location, severity, duration, and treatment requirements. Risks of haemostatic treatment, such as thrombosis, may also be different in patients with AHA who are often older and have cardiovascular risk factors. Here we report our experience with managing breakthrough bleeds in the GTH‐AHA‐EMI study with particular emphasis on dosing of haemostatic therapies and thromboembolic safety.

## 2 Methods

### 2.1 Study Oversight

This study was a post hoc analysis of data from the multicentre, open‐label, single‐arm, phase 2 GTH‐AHA‐EMI clinical trials (NCT04188639, registered at www.clinicaltrials.gov). The trial enrolled adult patients with AHA based on a reduced FVIII activity and a detectable anti‐FVIII inhibitor. Patients with congenital haemophilia were excluded. Conditions expected to increase the thromboembolic risk were also excluded, for example known antiphospholipid antibodies, other severe acquired or congenital forms of thrombophilia, or treatment with APCC. FVIII activity was measured in local laboratories and the central laboratory using a bovine chromogenic assay. Patients received emicizumab for bleed prophylaxis (6 and 3 mg/kg on days 1 and 2, respectively, followed by 1.5 mg/kg weekly until week 12). Immunosuppression was withheld for the first 12 weeks of study. Breakthrough bleeds during prophylaxis with emicizumab were treated at the investigator's discretion, including the choice of haemostatic agent, dosing, and use of concomitant tranexamic acid (TA). The protocol only advised against the use of APCC.

The primary endpoint was the number of CRNB during the 12 weeks of treatment with emicizumab. Bleeding was defined as clinically relevant if it required intervention by a healthcare professional or caused pain or any kind of disturbance of the patient's daily life. To differentiate major from non‐major bleeding, International Society on Thrombosis and Haemostasis (ISTH) criteria were applied. Detailed information on endpoints and major findings of this study has been published [10].

All patients gave written consent before enrolment and all procedures were in accordance with the International Conference on Harmonization Guidelines for Good Clinical Practice and were approved by the regulatory authorities in Germany and Austria and by the ethics committees of all participating centres.

### 2.2 Data Acquisition

The following information was retrieved from the original study database: clinical and laboratory data at baseline and follow‐up visits, start and duration of bleeding events, haemostatic drugs with starting dose and interval, treatment duration, and total dose. Information on treatment‐emergent adverse events was also retrieved from the study database. To gain further insight into circumstances and management of bleeds, we created a questionnaire that was answered by the participating centres for all 22 CRNB. This questionnaire assessed the WHO performance status at the time of bleeding, information on physical activity, reasons for bleeding, laboratory data, information on interventions needed to treat bleeding (including endoscopy, surgery, and endovascular procedures), intensive care unit admission, and outcomes and sequelae of bleeds.

### 2.3 Statistical Data Analysis

Data were reported in tabulated form and aggregated using median, interquartile range, and range as appropriate. A Wilcoxon non‐parametric test was used to compare rFVIIa doses between patient groups.

## 3 Results

### 3.1 Characteristics of CRNB

During the first 12 weeks of prophylaxis with emicizumab in patients with newly diagnosed AHA, 22 CRNB occurred in 14 of 47 patients (Table 1). Thirteen of those bleeds were classified as clinically relevant non‐major (CRNM), nine were classified as clinically relevant major (CRM). Six bleeds were mucosal, five musculoskeletal, four thoracic, four subcutaneous, two intraabdominal after surgery and one intracranial. The bleeds occurred between 1 and 47 days after starting emicizumab (median 35 days; IQR 11–53 days). Four bleeds occurred during the first 4 days of prophylaxis with emicizumab, most likely before steady‐state drug concentration was reached. The duration of the bleeds varied between 2 and 50 days (median 9 days; IQR 4–20 days). Two thirds of the bleeds occurred spontaneously (15/22), six were related to trauma or surgery, one related to infection.

**TABLE 1:** Overview of CRNB during the first 12 weeks of emicizumab prophylaxis.

Patient ID	Bleed number	Begin of bleed (day)	Duration of bleed (days)	Severity	Treatment	Location	Bleed type	Outcome
ID‐01	1	11	8	CRNM	No	Upper extremity, muscle	Spontaneous	Resolved
ID‐02	2	54	9	CRM	Yes	Abdomen, gastrointestinal	Spontaneous	Permanent harm^*^
ID‐02	3	74	2	CRM	Yes	Retroperitoneal	Spontaneous	Death
ID‐03	4	35	50	CRNM	No	Upper extremity, skin	Spontaneous	Resolved
ID‐04	5	2	10	CRNM	Yes	Upper extremity, muscle	Spontaneous	Resolved
ID‐05	6	72	14	CRNM	Yes	Upper extremity, skin	Traumatic	Resolved
ID‐06	7	36	9	CRM	Yes	Abdomen, anal	Spontaneous	Resolved
ID‐06	8	36	2	CRNM	Yes	Abdomen, vaginal	Spontaneous	Resolved
ID‐07	9	37	10	CRNM	Yes	Thorax, hematoma	Spontaneous	Resolved
ID‐07	10	1	5	CRNM	Yes	Upper extremity, skin	Spontaneous	Resolved
ID‐08	11	18	22	CRM	Yes	Head, intraventricular	Traumatic	Resolved
ID‐08	12	34	21	CRNM	Yes	Abdomen, macrohematuria	Other (infection and manipulation)	Resolved
ID‐09	13	4	3	CRNM	Yes	Upper extremity, muscle	Related to procedure / surgery	Resolved
ID‐10	14	32	19	CRM	Yes	Torso, muscle	Traumatic	Resolved
ID‐11	15	53	4	CRM	Yes	Abdomen, intraabdominal	Related to procedure / surgery	Resolved
ID‐11	16	57	32	CRM	Yes	Abdomen, gastrointestinal	Spontaneous	Resolved
ID‐12	17	30	2	CRNM	Yes	Thorax, pleural effusion	Spontaneous	Permanent harm^**^
ID‐12	18	14	15	CRNM	Yes	Thorax, pleural effusion	Spontaneous	Permanent harm^**^
ID‐12	19	45	5	CRNM	Yes	Thorax, pleural hematoma	Spontaneous	Resolved
ID‐13	20	1	6	CRNM	Yes	Upper extremity, skin	Spontaneous	Resolved
ID‐13	21	9	4	CRM	Yes	Abdomen, intraabdominal	Related to procedure / surgery	Resolved
ID‐14	22	55	39	CRM	Yes	Abdomen, anal	Spontaneous	Resolved

*Note*: Begin: begin of bleed in days after initiating emicizumab; Duration: duration of bleed in days after start of bleed; CRM: clinically relevant major; CRNM: clinically relevant non‐major. A bleed was considered clinically relevant if it required intervention by a healthcare professional or if it caused pain or any kind of disturbance of the patient's daily life. Location: body region is provided first, then type or tissue of bleed second.

*Note*: A bleed was considered major or non‐major according to ISTH criteria (fatal bleeding, fall of Hb ≥ 2 g/dL, transfusion ≥ 2 units of RBCs, bleeding at a critical site).

*pseudoaneurysm.

**persistent dyspnoea.

### 3.2 Circumstances of Bleeding

At the time of bleeding most patients had a poor WHO performance status (WHO‐PS 2 or worse in 90% of occasions; WHO‐PS 3 or worse in 60%, Table 2). Patients were still bedbound at the time of most bleeding events. No bleeding event was related to physical exercise (physiotherapy, active sport, or work).

**TABLE 2:** Clinical circumstances at the time of bleeding.

			Physical activity							FVIII activity, bovine chromogenic assay (IU/dl)		Inhibitor (BU/ml)	
Patient ID	Bleed number	WHO PS	Active in sports	Working	Bedbound	Haemoglobin (g/dl)	Platelet count (/nl)	APTT (sec)	Fibrinogen (g/l)	At onset of bleeding	At last study visit before bleeding	At last study visit before bleeding	Emicizumab (µg/ml)
ID‐01	1	2	no	no	no	9	491	26	na	<0.4	<1	154.2	18.9
ID‐02	2	2	no	no	no	7.5	180	28	3.9	1.5	<1	544.3	46.3
ID‐02	3	4	no	no	yes	8.6	216	31	3.3	68	<1	544.3	46.3
ID‐03	4	1	no	no	no	na	na	na	na	na	2	16.9	31.7
ID‐04	5	3	no	no	yes	8	602	49	7.1	na	12	29.7	<10
ID‐05	6	2	no	no	no	10.6	436	38	na	na	3	8.7	19.0
ID‐06	7	4	no	no	yes	7.1	288	44	2	na	10	14.8	14.6
ID‐06	8	4	no	no	yes	7.1	288	44	2	na	10	14.8	14.6
ID‐07	9	2	no	no	yes	na	na	na	na	na	3	2.5	16.9
ID‐07	10	2	no	no	yes	11.8	323	63	5.1	<10	2.1	1.8	<10
ID‐08	11	3	no	no	yes	8.8	133	26	na	0.2	<1	8.7	14.8
ID‐08	12	3	no	no	yes	9.4	150	27	4.8	0	<1	140.9	12.9
ID‐09	13	1	no	no	no	10.3	359	30	3.2	13.4	23.5	0.9	<10
ID‐10	14	2	no	no	no	8	190	28	4.9	1.6	1.6	262.4	27.5
ID‐11	15	3	no	no	yes	8.2	338	42	3.5	<0.5	<1	102.2	15.9
ID‐11	16	4	no	no	yes	6.4	158	50	4.5	1.3	<1	102.2	15.9
ID‐12	17	3	no	no	yes	na	na	na	na	na	9.2	7.4	20.6
ID‐12	18	3	no	no	yes	na	na	33	4.9	7.5	10.9	4.3	21.5
ID‐12	19	na	na	na	na	na	na	na	na	na	9.2	7.4	20.6
ID‐13	20	4	no	no	yes	6.9	358	90	4.9	na	3	3.6	<10
ID‐13	21	4	no	no	yes	5.6	1136	43	4.6	na	50	7.3	<10
ID‐14	22	na	na	na	na	16.2	197	18	3.5	<1	<1	268.4	20.5

*Note*: This additional data was collected using a questionnaire that was answered by the participating centres for all 22 CRNB, except for the FVIII activity, inhibitor, and emicizumab level at last study visit before bleeding, which were the central laboratory measurements retrieved from the study database.

Abbreviations: APTT: activated partial thromboplastin time; FVIII: Factor VIII; na: not available; WHO PS: World Health Organization Performance Status.

Most often patients had haemoglobin levels <10 g/dl at the time of bleeding. Only four bleeding events were reported with a haemoglobin level >10 g/dl and just one of them was major. Platelet counts and fibrinogen levels were either normal or increased at the time of bleeding.

FVIII activity was often unknown at the time when patients presented with bleeding. In 10 of 12 events with known FVIII activity, it was reduced to less than 10 IU/dl. Exceptions were a patient who developed a new bleed under treatment with rpFVIII (ID‐02, event 3; FVIII 68 IU/dl) and a patient who had only moderately reduced FVIII activity and low inhibitor titre throughout the study (ID‐09, event 13; FVIII 13.4 IU/dl). Table 2 also reports FVIII activity measured in the central laboratory at the last visit before the onset of bleeding, suggesting that FVIII was presumably low in most cases. A notable exception was a case with postoperative bleeding (ID‐13, event 21; FVIII 50 IU/dl).

In the majority of bleeding events, the activated partial thromboplastin time (APTT) was normal as expected under treatment with emicizumab. An APTT prolongation of >40 s was reported in eight events. This included 3 events occurring in the first days of emicizumab prophylaxis (ID‐13 on day 1, ID‐07 on day 1, ID‐04 on day 2), when drug levels were presumably low. In the remaining five events, emicizumab levels measured during the previous study visit were also below the mean reported steady state level, ranging between <10 µg/ml and 15.9 µg/ml. In two of these events occurring in the same patient (ID‐11), low levels and APTT prolongation were explained by withdrawal of emicizumab and plasmapheresis 2 weeks earlier, due to mesenteric ischemia that was initially interpreted as a thromboembolic event but later explained by cholesterol embolization. In another case, prolonged APTT and low levels of emicizumab were related to serious bleeding and surgery, which may have contributed to faster clearance of the drug (ID‐13). The remaining two events occurring in the same patient (ID‐06), APTT prolongation and low levels of emicizumab had no clear reason. Anti‐drug antibodies and lack of compliance were excluded.

### 3.3 Treatment

20 out of 22 bleeds required some form of treatment, including rFVIIa, rpFVIII, factor XIII (FXIII), fibrinogen concentrate, red blood cell concentrates (RBC), platelet concentrates (PC), fresh frozen plasma (FFP), TA, or vitamin K. The two bleeds not requiring haemostatic treatment were non‐major. 17 of 20 bleeds were treated with bypassing agents or rpFVIII Table 3). Detailed dosing information on all blood products and TA is provided in Table 4.

**TABLE 3:** Use of bypassing agents and rpFVIII for CRNB under emicizumab.

Patient ID	Bleed no	Drug	Starting dose		Starting interval	Duration of treatment	Total dose	TA
ID‐02	2	rFVIIa	6 mg	67 µg/kg	Q3H	9	360 mg	yes
ID‐02	3	rpFVIII	8000 IU	89 IU/kg	Continuously	2	19,872 IU	no
ID‐04	5	rpFVIII	1000 IU	10 IU/kg	QD	1	1000 IU	no
ID‐04	5	rFVIIa	9 mg	90 µg/kg	QD	4	36 mg	yes
ID‐06	7	rFVIIa	5 mg	98 µg/kg	Q3H	8	150 mg	yes
ID‐07	9	rFVIIa	6 mg	83 µg/kg	Q4H	3	48 mg	no
ID‐07	10	rFVIIa	7 mg	97 µg/kg	Q4H	2	63 mg	no
ID‐08	11	rFVIIa	6 mg	68 µg/kg	Q6H	16	294 mg	no
ID‐08	12	rFVIIa	6 mg	68 µg/kg	BID	8	60 mg	no
ID‐09	13	rFVIIa	7 mg	80 µg/kg	QD	1	7 mg	yes
ID‐11	15	rFVIIa	5 mg	96 µg/kg	PRN	4	60 mg	no
ID‐11	16	rFVIIa	5 mg	96 µg/kg	PRN	32	465 mg	no
ID‐12	17	rFVIIa	5 mg	74 µg/kg	QD	1	5 mg	no
ID‐12	18	rFVIIa	6 mg	88 µg/kg	QD	1	6 mg	no
ID‐12	19	rFVIIa	5 mg	74 µg/kg	QD	1	5 mg	no
ID‐13	20	rFVIIa	6 mg	86 µg/kg	Q4h	6	138 mg	no
ID‐13	21	rFVIIa	6 mg	86 µg/kg	PRN	5	156 mg	no
ID‐13	21	rpFVIII	14,000 IU	200 IU/kg	Bolus followed by continuous infusion	2	n/a	no
ID‐14	22	rFVIIa	5 mg	100 µg/kg	PRN	15	230 mg	no

*Note*: Drug: only referring to recombinant activated factor VII (rFVIIa) or recombinant porcine factor VIII (rpFVIII); QD: once daily; BID: twice daily; Q6H: every six hours; Q4H: every four hours; Q3H: every three hours; PRN: pro re nata; TA: tranexamic acid. Duration of treatment in days.

**TABLE 4:** Detailed haemostatic treatment and blood product support for CRNB under emicizumab.

Patient ID		Bleed number	Drug	Dosing information and interval	Number of doses	Total dose
ID‐02	2 2 2 2		RBC rFVIIa TA FFP	d4–9: 1 unit PRN d1–5: 6 mg Q3H d5–9: 8 mg Q3H d5–9: 1000 mg Q8H d5–9: 1 unit PRN	21 28 24 12 16	21 units 168 mg 192 mg 12,000 mg 16 units
	3 3 3 3		RBC FFP rpFVIII FXIII	d1–2: 1 unit PRN d2: 1 unit PRN d1–2: continuous infusion d2: 2500 QD	7 2 na 1	7 units 2 units 19,872 units 2500 units
ID‐04	5 5 5		rpFVIII rFVIIa TA	d1: 1000 IU QD d1–4: 9 mg QD d2–10: 1000 mg Q8H	1 4 27	1000 units 36 mg 27,000 mg
ID‐05	6		TA	d11–16: 1000 mg Q8H	15	15,000 mg
ID‐06	7&8 7 7 7 7 7		RBC rFVIIa FXIII FFP TA Vitamin K	d2–5: 1 unit PRN d2–9: 5 mg variable intervals d2: 1250 units PRN d2: 1 unit PRN d5–9: 1000 mg Q8H d2, d7‐8: 10 mg QD	4 30 2 4 15 3	4 units 150 mg 2500 units 4 units 15,000 mg 30 mg
ID‐07	9		rFVIIa	d4–6: 6 mg variable intervals	8	48 mg
	10		rFVIIa	d2–3: 7 mg Q4H	9	63 mg
ID‐08	11		rFVIIa	d1–7: 6 mg Q6H d8–12: 6 mg Q8H d13–16: 6 mg BID	26 15 8	156 mg 90 mg 48 mg
	12		rFVIIa	d2: 6 mg BID d3–5: 6 mg QD d6–7: 6 mg BID d8: 6 mg BID	2 3 3 2	12 mg 18 mg 18 mg 12 mg
ID‐09	13 13		rFVIIa TA	d2: 7 mg QD d1–3: 1000 mg Q8H	1 9	7 mg 9000 mg
ID‐10	14 14		RBC TA	d5 and 8: 2×2 units d15: 2 units d5: 1000 mg QD d6–10: 1000 mg Q8H d11: 1000 mg BID d12–19: 1000 mg Q8H	4 1 1 15 2 24	8 units 2 units 1000 mg 15,000 mg 2000 mg 24,000 mg
ID‐11	15 15&16 15&16 16 16 16 16 16		rFVIIa Vitamin K RBC rFVIIa PC FXIII Fibrinogen Vitamin K	d1–4: 5 mg PRN d4(bl.15)–d7(bl.16): 2 mg weekly d1(bl.15)–d32(bl.16): 1 unit PRN d1–32: 5 mg PRN d32: 1 unit QD d30: 2500 units QD d21: 2000 mg QD d10–31: 10 mg weekly	12 2 26 93 1 1 1 4	60 mg 4 mg 26 units 465 mg 1 unit 2500 units 2000 mg 40 mg
	17		rFVIIa	d1: 5 mg QD	1	5 mg
ID‐12	18		rFVIIa	d1: 6 mg QD	1	6 mg
	19		rFVIIa	d1: 5 mg QD	1	5 mg
ID‐13	20 20 20&21 21 21 21		rFVIIa Vitamin K RBC rFVIIa rpFVIII rpFVIII	d2–7: 6 mg (Q4H d2, Q6H d3‐7) d1–2: 10 mg QD d5–11: 1 unit PRN d1–5: 6 mg PRN d4: 14,000 IU Bolus d4–5: continuous infusion	23 2 10 26 1 N/A	138 mg 20 mg 10 units 156 mg 14,000 units N/A
ID‐14	22		rFVIIa	d2–16: 5 mg PRN	46	230 mg

*Note*: 20 of 22 CRNB were treated. Day 1 (d1) means first day of bleed.

Abbreviations: TA, tranexamic acid; rFVIIa, recombinant activated factor VII; rpFVIII, recombinant porcine factor VIII; FXIII, factor XIII; RBC, red blood cell concentrate; PC, platelet concentrate; FFP, fresh frozen plasma; PRN, pro re nata; Q3H, every three hours; Q4H, every four hours; Q6H, every six hours; Q8H, every eight hours; na, not available; IU, international units.

**FIGURE 1:**
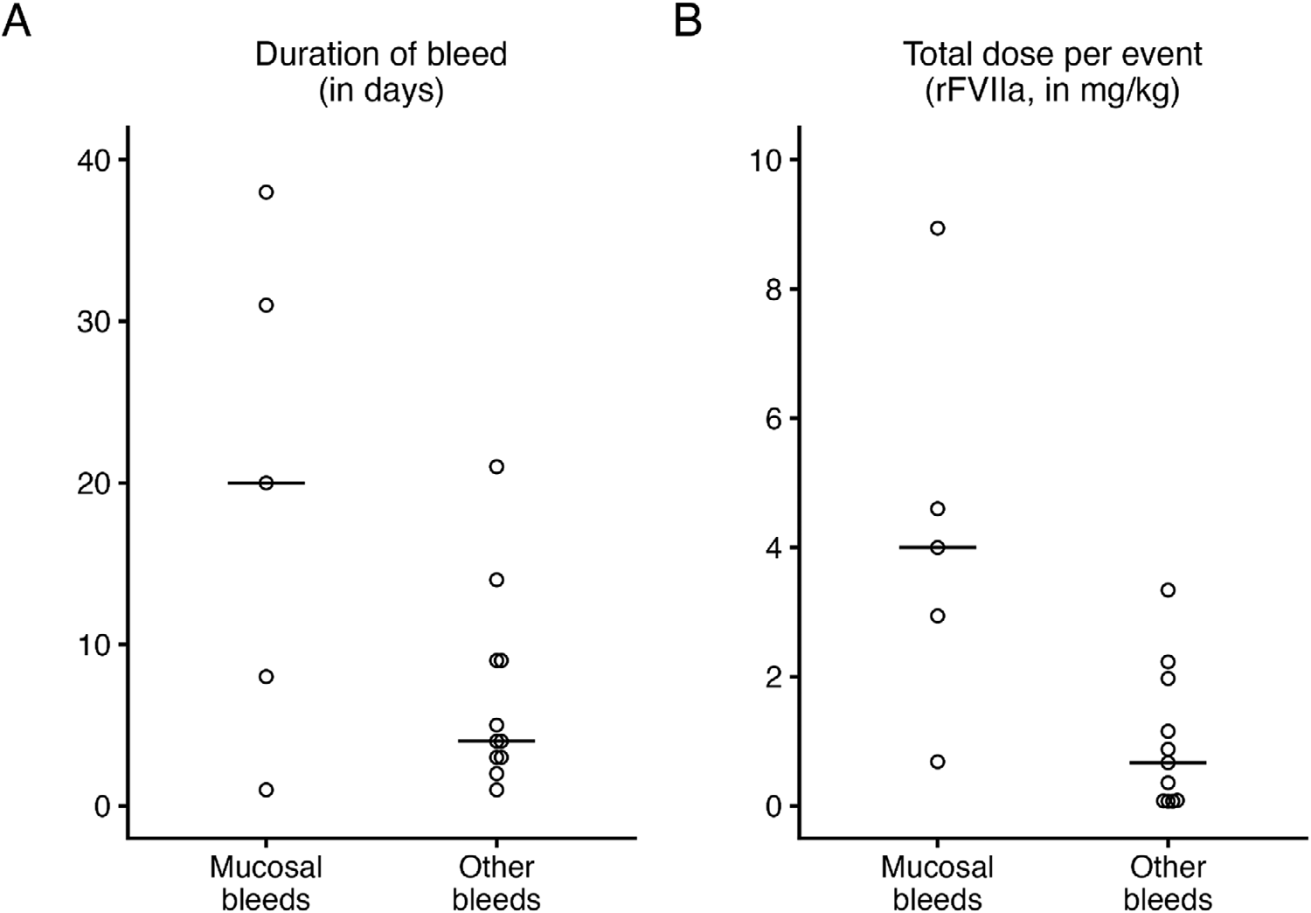
Bleeding episodes treated with rFVIIa (*n* = 16), comparing mucosal bleeds (*n* = 5) and non‐mucosal bleeds (*n* = 11). (A) Bleed duration (days). (B) Cumulative body weight—adjusted rFVIIa dose.

rFVIIa was the most frequently used agent (16 events). Starting doses chosen by investigators ranged between 5–9 mg (67–100 µg/kg body weight) per dose, starting intervals varied between once daily and every 3 h (Table 3). The total duration of treatment varied from 1–32 days. The median total dose per event was 61.5 mg (range 5–465 mg; IQR 21.5–193 mg). Among the 10 patients receiving rFVIIa for 16 CRNB occurring during weeks 1 and 12 of emicizumab therapy, the median total dose per patient was 190 mg (range 7–525 mg; IQR 36–354 mg). Body weight‐adjusted dosing was higher for mucosal bleeds (median 4 mg/kg, range 0.68–8.94 mg/kg, *n* = 5) compared with other bleeds (median 0.67 mg/kg, range 0.07–3.34 mg/kg, *n* = 11, *p* = 0.017). This was accompanied by longer bleed duration in mucosal cases (median 20 days, range 1–38 days) versus other bleeds (median 4 days, range 1–11 days, Figure 1). In four events, TA was given together with rFVIIa.

rpFVIII was used in three cases with starting doses ranging between 1000 IU–14,000 IU. Treatment duration ranged between one and two days.

FXIII was used additionally in three particularly severe events. These events occurred were in the iliopsoas muscle (ID‐02, event 3), rectum (ID‐06, event 7), or the upper gastrointestinal tract (ID‐11, event 16). They occurred spontaneously, were classified as major and required 4–27 units of RBC. The patients needed support at the intensive care unit, and one died due to multiorgan failure.

TA was used in six events. It was used either in combination with rFVIIa (4 events) or alone (2 events). These bleeds were intramuscular (three‐sixth), gastrointestinal (two‐sixth), or cutaneous (one‐sixth). The median duration of treatment with TA was 6 days (IQR 5–11 days). The median total dose was 15 g (IQR 11–31 g) per event.

### 3.4 Outcomes

Overall, eight bleeds required hospital admission, 13 caused an extension of hospitalization and 12 needed an invasive intervention and/or required treatment at the intensive care unit (Table 5). At the end of the observation period of 12 weeks, nearly all bleeds were reported as resolved.

**TABLE 5:** Consequences and complications of CRNB and haemostatic treatment.

	Clinically relevant non‐major (*n *= 13)	Clinically relevant major (*n *= 9)
**Consequences and complications of bleeding**		
Admission to hospital, *n* (%)	4 (31)	4 (44)
Extension of hospitalization, *n* (%)	6 (46)	7 (78)
Treatment with haemostatic drug, *n* (%)	11 (85)	9 (100)
Transfusion of erythrocyte or platelet concentrate, *n* (%)	2 (15)	6 (67)
Need for invasive intervention or ICU, *n* (%)	4 (31)	8 (89)
Permanent harm caused by bleeding, *n* (%)	2 (15)	1 (11)
Death caused by bleeding, *n* (%)	0 (0)	1 (11)
**Consequences and complication caused by haemostatic treatment**		
Allergic reaction, *n* (%)	0 (0)	0 (0)
Thromboembolic events, *n* (%)	0 (0)	0 (0)
Permanent harm caused by haemostatic treatment, *n* (%)	0 (0)	0 (0)
Death caused by haemostatic treatment, *n* (%)	0 (0)	0 (0)

*Note*: Treatment with haemostatic drug means any other than emicizumab, including tranexamic acid, recombinant activated factor VII, recombinant porcine factor VIII and factor XIII.

Permanent harm from bleeding events was reported in two cases. First, ID‐12 had three bleeds related to thoracentesis for pleural effusion. The first puncture was covered with rFVIIa and removed 1500 mL of clear fluid. One week later another puncture was required and removed 1600 mL haemorrhagic fluid. This event was counted as a bleed. The patient underwent another puncture and a video‐assisted thoracoscopy. At final follow up, he reported residual respiratory distress, which was considered as permanent harm from bleeding. Second, ID‐02 had a particularly serious course starting with a spontaneous gastrointestinal bleed from Mallory‐Weiss syndrome on day 54 (event 2). Several invasive interventions were necessary to control the bleed, including endoscopic treatment and repeated endovascular coiling. The patient developed a pseudoaneurysm at the external iliac artery access site that was classified as permanent harm. One week later, the patient developed multiple retroperitoneal haematomas (event 3). Despite treatment with rpFVIII and other haemostatic drugs, this bleed could not be stabilized and the patient died of multiorgan failure in haemorrhagic shock on the intensive care unit.

### 3.5 Safety of Haemostatic Treatment

No thromboembolic or other adverse event was reported in relation to haemostatic treatment of CRNB. The two reported thromboembolic events occurring during the study (one thrombophlebitis in week one, one stroke in week 20) were not related to the treatment of bleeds.

## 4 Discussion

The current study provides a detailed analysis of 22 bleeds occurring in the GTH‐AHA‐EMI study during 12 weeks of prophylaxis with emicizumab. Four bleeds occurred very early, when emicizumab concentrations were not yet in steady state. Six bleeds were related to trauma or surgery and two occurred after emicizumab had been withdrawn and removed from circulation by plasmapheresis. However, it should be noted that most bleeds were spontaneous, and many occurred after completing the first week of emicizumab, when steady state concentrations of the drug are already achieved. Therefore, prevention and management of breakthrough bleeds in AHA patients receiving emicizumab is a relevant task.

When haemostatic therapy was required, rFVIIa (*n* = 16) and rpFVIII (*n* = 3) were the preferred medications. Dosing was usually in line with guideline recommendations and licensed summary of product characteristics. This was overall safe and effective.

rFVIIa was the most frequently used haemostatic drug. The median total dose of rFVIIa was 190 mg (IQR 36–354 mg) per patient, which is only slightly lower compared to the median cumulative dose of 210 mg (IQR 56–491 mg) reported in AHA patients not receiving emicizumab in the GTH‐AH 01/2010 study [16]. This finding is somewhat unexpected, as one of the benefits of emicizumab discussed in the literature is to reduce the need for other haemostatic treatments [17]. However, this comparison should be interpreted with caution: only 10 of 47 patients in the current study received rFVIIa for clinically relevant non‐major bleeding (CRNB) (and 3 received rpFVIII), whereas 63 of 102 patients in the GTH‐AH 01/2010 study received rFVIIa (and none received rpFVIII). These data suggest that, at the population level, emicizumab may reduce overall rFVIIa consumption, despite similar per‐patient cumulative doses among treated individuals. In other words, emicizumab is generally associated with a lower occurrence of CRNBs, but when a breakthrough bleeding event occurs, patients seem to require similar amounts of haemostatic agents as compared with patients not on emicizumab.

One possible explanation for the observed dosing need is the distribution of bleed types in the study. Mucosal bleeding was the most frequent bleed category in the current study and these bleeds tended to require higher cumulative doses of rFVIIa. In fact, 10 of the 20 treated bleeds had a duration of >10 days and this included five mucosal bleeds. Mucosal bleeds often pose a more complex therapeutic challenge than subcutaneous or musculoskeletal bleeds, which represent the predominant bleeding phenotype in patients with AHA who are not receiving emicizumab [3, 5, 16, 18). It is reassuring to note that there were no thromboembolic events despite the combination of emicizumab with the licensed dose of rFVIIa, in many cases over a prolonged period and in combination with TA.

We paid special attention toward the safety of TA, as TA together with rFVIIa resulted in two fatal strokes (2 out of 21 patients receiving this combination) in the GTH‐AH 01/2010 study [19]. In the current study, the combination of TA with rFVIIa and emicizumab was used in 4 events and was not associated with thromboembolic complications.

Our analysis adds to the limited literature about the use of emicizumab in patients with AHA. Systematic reviews were done by Pasca et al. [9] (reporting 73 patients, data cut December 2022) and Ikbel et al. [18] (reporting 171 patients, data cut August 2024). The latter review also includes the patients from the AGEHA [11] and GTH‐AHA‐EMI [10] trial. Among these 171 patients, three cases of deep vein thrombosis and two cases of stroke were reported, corresponding to an event rate of approximately 2.9%.

We searched the literature for additional case reports and series published after these systematic reports. One reported the course of an elderly woman with AHA resistant to immunosuppressive therapy who was treated with emicizumab over four years without major bleeding or thromboembolic complications, despite the need for emergency surgery with concomitant use of rFVIIa [20].

In a retrospective assessment of a single‐centre cohort of 22 adults with AHA, one 26‐year‐old female patient received emicizumab for severe refractory bleeding six month postpartum [21]. She had received rFVIIa and FFP before starting emicizumab, but not thereafter. No thrombotic event occurred.

Two case reports addressed the complicated management due to underlying malignancies and comorbidities. Wolff et al. [22] published a series of 11 patients who developed AHA due to immune checkpoint inhibitors. One of them, a 68‐year‐old male with metastatic melanoma, was treated with emicizumab after presentation with diffuse ecchymosis. Resolution of bleeding was achieved, and no thrombotic event occurred. Cuschera et al. [23] reported a difficult case of AHA secondary to multiple myeloma in a 76‐year‐old woman. She received emicizumab in combination with methylprednisolone as first line treatment. No other haemostatic treatment was necessary. Shortly after a relapse of her myeloma was diagnosed and myeloma‐specific therapy was started. In the further course, she developed therapy‐associated thrombocytopenia and atrial fibrillation requiring low dose apixaban. Despite the many bleeding and thromboembolic risk factors coexisting in this patient, she had no bleeding or thromboembolic complications'; illustrating that emicizumab represents a valuable therapy option for complex AHA patients.

Kishi et al. [24] compared clinical characteristics and outcomes before and after emicizumab approval for AHA in Japan. In the seven AHA patients receiving emicizumab, no thromboembolic events were recorded, including those patients where it was combined with rFVIIa.

Poston et al. [25] reported insights from a cohort of 62 AHA patients treated with emicizumab in the United States. Six breakthrough bleeds occurred that were treated with rFVIIa, three required additional haemostatic treatment including FVIII, rpFVIII and tranexamic acid. The authors reported that vascular risk factors were present in 63% of patients. Two patients experienced thrombotic events during treatment with emicizumab. These events were not attributed to emicizumab itself but rather to dispositional risk factors. One patient had atrial fibrillation and developed a deep vein thrombosis during cessation of previous anticoagulant therapy. The other patient suffered a stroke while antiplatelet therapy was withheld.

Pastore et al. [26] compared the use of emicizumab (*n* = 10) to a historic cohort not using emicizumab (*n* = 11). rFVIIa was required in six patients of both cohorts with no thrombotic event reported. A further case report of an 82‐year‐old woman with several comorbidities and multiple risk factors reported a favourable outcome with no bleeds or thrombotic events after starting emicizumab [27].

Summarizing these reports of AHA patients receiving emicizumab, a total of nine thromboembolic events (five events of deep vein thrombosis, four strokes) were reported in 255 patients. This rate of 3.6% appears low considering the age, acute illness, and additional risk factors in these patients. In several cases, the authors considered a causal relationship with emicizumab to be unlikely.

Our study adds to the growing body of evidence for the thromboembolic safety of emicizumab in AHA patients by providing more detail about the haemostatic medication given to patients on emicizumab. The two events reported in the GTH‐AHA‐EMI study (one thrombophlebitis in week one, one ischemic stroke in week 20, while emicizumab was still given) were not related to additional haemostatic medication for bleeding. Adding these events to the events reported in the literature, a total of 11 thromboembolic events occurred in 302 treated patients (3.6%). This rate is similar to the rate reported for rFVIIa in a systematic literature review [28].

Some limitations of our study should be considered. First, FVIII activity and emicizumab concentrations were sometimes not available at the time of bleeding. It should be noted that peak thrombin generation measured early after emicizumab loading was related to the risk of breakthrough bleeding in this study [15], but samples were unavailable to repeat this measurement at the time of bleeding. Second, participation in a clinical trial could have shifted the investigator's behaviour with regards to hospital admission and treatment of bleeding events under emicizumab. At the time the study was conducted, experience in treating bleeds in AHA patients receiving emicizumab was limited, which may have prompted more caution in clinical decisions. In routine practice today, with increasing evidence supporting successful bleed management under emicizumab, outpatient treatment and potentially shorter use of additional haemostatic agents might be more common. Third, comparisons with historic data from the pre‐emicizumab era—including the GTH‐AH 01/2010 study—are challenging due to changes in treatment standards over time. This applies not only to immunosuppressive strategies but also to haemostatic management, as, for example, rpFVIII was not approved prior to 2015. Still, we believe that the current report is valuable for physicians treating breakthrough bleeds in AHA patients under emicizumab by reassuring the efficacy and thromboembolic safety of the dosing regimens administered here.

## 5 Conclusion

In conclusion, haemostatic therapy for CRNB under emicizumab was generally effective, with rFVIIa being the most frequently used agent, often administered at variable dosing schedules over multiple days. Although most bleeds resolved, serious morbidity occurred in two of 47 patients. No thromboembolic complications were observed, indicating that the haemostatic strategies used in this cohort appeared safe. Clinical care should continue to focus on minimizing the risk of breakthrough bleeding in AHA patients receiving emicizumab.

## Author Contributions

*Study conception, data analysis, and manuscript writing*: Halet Türkantoz, Andreas Tiede. *Data acquisition*: Halet Türkantoz, Andreas Tiede, Richard Greil, Christina Hart, Ulrich Sachs, Robert Klamroth, Paul Knöbl, Christian Pfrepper, Johannes Oldenburg, Karolin Trautmann‐Grill, Christiane Dobbelstein, Inga M Schimansky. *Manuscript and approval of its final version*: all authors. Andreas Tiede and Halet Türkantoz had access to the raw data and ensured the completeness and accuracy of all data and analyses.

## Funding

The GTH‐AHA‐EMI study was sponsored by Gesellschaft für Wissens‐ und Technologietransfer (GWT‐TUD) and received funding from Hoffman‐La Roche.

## Ethics Statement

All procedures were in accordance with the Declaration of Helsinki and the International Conference on Harmonization Guidelines for Good Clinical Practice and were approved by the regulatory authorities in Germany and Austria and by the ethics committees of all participating centres. All patients gave written consent before enrolment.

## Conflicts of Interest

HT has no conflict of interest to disclose. CD reports honoraria for lectures or consultancy and travel grants from Chugai, Roche and SOBI. RG reports institutional grants for research and studies from Celgene, Roche, Merck, Takeda, AstraZeneca, Novartis, Amgen, BMS, MSD, Sandoz, Abbvie, Gilead, and Daiichi Sankyo, and honoraria for lectures or consultancy from Celgene, Roche, Merck, Takeda, AstraZeneca, Novartis, Amgen, BMS, MSD, Sandoz, Abbvie, Gilead, Daiichi Sankyo, and Sanofi. CH reports honoraria for lectures or consultancy from Bayer, SOBI, Roche, BMS, Pfizer, Novo Nordisc, Daiichi‐Sankyo, Novartis, and Sanofi. CH reports institutions grants for research from Sobi. RK reports institutional grants for research and studies from Bayer, CSL Behring, Novo Nordisk, Octapharma, and SOBI, and honoraria for lectures or consultancy from Bayer, Biotest, Biomarin, CSL Behring, Grifols, LFB, Novo Nordisk, Octapharma, Pfizer, Roche/Chugai, Sanofi, SOBI, and Takeda. PK reports institutional grants for research and studies from Ablynx/Sanofi, Novo Nordisk, Roche, and Takeda, and honoraria for lectures or consultancy from Ablynx/Sanofi, Alexion, Biotest, CSL Behring, Novo Nordisk, Roche, Takeda, and Technoclone. JO reports institutional grants for research and studies from Bayer, Biotest, CSL Behring, Novo Nordisk, Octapharma, Pfizer, SOBI and Takeda, and honoraria for lectures or consultancy from Bayer, Biomarin, Biotest, Chugai, CSL Behring, Grifols, LFB, Novo Nordisk, Octapharma, Pfizer, Roche, Sanofi, SOBI, and Takeda. CP reports institutional grants for research and studies from Chugai/Roche, Takeda, Zacros, and Leo Pharma, and honoraria for lectures or consultancy from Bayer, Biomarin, Chugai/Roche, CSL Behring, Novo Nordisk, Pfizer, BMS, SOBI, and Takeda. UJS reports institutional grants for research and studies from Octapharma, and honoraria for lectures or consultancy from Bayer, SOBI, CSL Behring, and Pfizer. IMS reports honoraria and travel grants from Novo Nordisk. KTG reports honoraria for lectures or consultancy from Takeda and Roche, SOBI, Novo Nordisk, Sanofi, Grifols, Amgen and Novartis. CP reports institutional grants for research and studies from Chugai/Roche, Takeda, Zacros, and LeoPharma, and honoraria for lectures or consultancy from Bayer, Biomarin, Chugai/Roche, CSL Behring, Novo Nordisk, Pfizer, BMS, SOBI, and Takeda. AT reports institutional grants for research and studies from Bayer, Biotest, Chugai, Novo Nordisk, Octapharma, Pfizer, Roche, SOBI, and Takeda, and honoraria for lectures or consultancy from Bayer, Biomarin, Biotest, Chugai, CSL Behring, Novo Nordisk, Octapharma, Pfizer, Roche, SOBI, and Takeda.

## Statements Relating to the Ethics and Integrity Policies

This work has not been published and is not being considered for publication elsewhere.

## Permission to Reproduce Material From Other Sources

Not applicable

## Data Availability

Data supporting the findings of this study are available from the corresponding author upon reasonable request.
